# Oral Furosemide and Hydrochlorothiazide/Amiloride versus Intravenous Furosemide for the Treatment of Resistant Nephrotic Syndrome

**DOI:** 10.3390/jcm12216895

**Published:** 2023-11-01

**Authors:** Georgiana Frățilă, Bogdan Marian Sorohan, Camelia Achim, Andreea Andronesi, Bogdan Obrișcă, Gabriela Lupușoru, Diana Zilișteanu, Roxana Jurubiță, Raluca Bobeică, Sonia Bălănică, Georgia Micu, Valentin Mocanu, Gener Ismail

**Affiliations:** 1 Department of Nephrology, “Carol Davila” University of Medicine and Pharmacy, 020021 Bucharest, Romania; valentina-georgiana.fratila@drd.umfcd.ro (G.F.); camelia.achim@umfcd.ro (C.A.); andreea.andronesi@umfcd.ro (A.A.); bogdan.obrisca@drd.umfcd.ro (B.O.); gabriela.lupusoru@umfcd.ro (G.L.); diana.zilisteanu@umfcd.ro (D.Z.); gener.ismail@umfcd.ro (G.I.); 2 Department of Kidney Transplantation, Fundeni Clinical Institute, 022328 Bucharest, Romania; 3 Department of Nephrology, Fundeni Clinical Institute, 022328 Bucharest, Romania; roxana_jurubita@yahoo.com (R.J.); ralbobeica@gmail.com (R.B.); sonia.balanica@gmail.com (S.B.); elenageorgia.micu@gmail.com (G.M.); valentin-dumitrel.mocanu@drd.umfcd.ro (V.M.)

**Keywords:** diuretic, furosemide, hydrochlorothiazide, amiloride, nephrotic syndrome, resistant, edema, oral, intravenous, weight

## Abstract

Background: Data on diuretic treatment in nephrotic syndrome (NS) are scarce. Our goal was to assess the non-inferiority of the combined oral diuretics (furosemide/hydrochlorothiazide/amiloride) compared to intravenous (i.v.) furosemide in patients with NS and resistant edema. Methods: We conducted a prospective randomized trial on 22 patients with resistant nephrotic edema (RNE), defined as hypervolemia and a FENa < 0.2%. Based on a computer-generated 1:1 randomization, we assigned patients to receive either intravenous furosemide (40 mg bolus and then continuous administration of 5 mg/h) or oral furosemide (40 mg/day) and hydrochlorothiazide/amiloride (50/5 mg/day) for a period of 5 days. Clinical and laboratory measurements were performed daily. Hydration status was assessed by bioimpedance on day 1 and at the end of day 5 after treatment initiation. The primary endpoint was weight change from baseline to day 5. Secondary endpoints were hydration status change measured by bioimpedance and safety outcomes (low blood pressure, severe electrolyte disturbances, acute kidney injury and worsening hypervolemia). Results: Primary endpoint analysis showed that after 5 days of treatment, there was a significant difference in weight change from baseline between groups [adjusted mean difference: −3.33 kg (95% CI: −6.34 to −0.31), *p* = 0.03], with a higher mean weight change in the oral diuretic treatment group [−7.10 kg (95% CI: −18.30 to −4.30) vs. −4.55 kg (95%CI: −6.73 to −2.36)]. Secondary endpoint analysis showed that there was no significant difference between groups regarding hydration status change [adjusted mean difference: −0.05 L (95% CI: −2.6 to 2.6), *p* = 0.96], with a mean hydration status change in the oral diuretic treatment group of −4.71 L (95% CI: −6.87 to −2.54) and −3.91 L (95% CI: −5.69 to −2.13) in the i.v. diuretic treatment group. We observed a significant decrease in adjusted mean serum sodium of −2.15 mmol/L [(95% CI: −4.25 to −0.05), *p* = 0.04]), favored by the combined oral diuretic treatment [−2.70 mmol/L (95% CI: −4.89 to −0.50) vs. −0.10 mmol/L (95%CI: −1.30 to 1.10)]. No statistically significant difference was observed between the two groups in terms of adverse events. Conclusions: A combination of oral diuretics based on furosemide, amiloride and hydrochlorothiazide is non-inferior to i.v. furosemide in weight control of patients with RNE and a similar safety profile.

## 1. Introduction

Edema is a common manifestation of nephrotic syndrome (NS). NS pathogenesis involves sodium and water retention, and pathogenic treatment consists of decongestion, where diuretic treatment plays an important role [1,2]. The two main hypotheses of edema, underfill and overfill, have led to different diuretic approaches, but guidelines on managing volume overload are missing due to a lack of randomized clinical trials (RCTs). While the underfill hypothesis offers some possible explanations for water and sodium retention, knowledge regarding the epithelium sodium channel (ENaC) and animal models of NS have provided new data on how certain filtered serine proteases specifically lead to sodium retention, according to the overfill hypothesis [3,4]. 

The underfill hypothesis exposes that proteinuria leads to hypoalbuminemia, resulting in reduced plasma oncotic pressure with a consequent leak of fluid into the interstitial space, which causes edema. This also causes a decrease in effective arterial blood volume with compensatory activation of the renin–angiotensin–aldosterone system (RAAS) and other neurohormonal responses (e.g., antidiuretic hormone [ADH], angiotensin II [AgII]), sympathetic nervous system [SNS] and atrial natriuretic peptide [ANP]) leading to secondary sodium and water retention and edema formation [5]. RAAS or SNS blockade have failed to properly increase natriuresis in these patients [6,7,8,9,10,11,12].

The potential of urinary proteases to act on different channels of the distal renal tubule, especially ENaC, was demonstrated in murine models of NS [13,14,15]. Activation of ENaC implies cleavage at specific sites of both alpha and gamma subunits within the extracellular domain. All of this knowledge about the theory of sodium retention has changed the optics of the diuretic approach. Thus, through its inhibitory activity on ENaC, but also through its ability to inhibit urokinase-type plasminogen activator (uPA) and thus reduce plasmin levels, amiloride may be the diuretic of choice in the treatment of nephrotic edema [16].

In addition to non-pharmacological interventions such as sodium and water restrictions, most clinicians would choose as their first treatment option a loop diuretic, most frequently furosemide. But still, many patients fail to properly decrease the extracellular fluid volume as they become diuretic resistant [17]. In this case, it is assumed that diuretic combination can overcome this. Data from patients with heart failure showed that diuretic combinations such as loop diuretic with a thiazide diuretic or with acetazolamide have proven their efficiency. Also, combined diuretics seem to be effective in patients with advanced chronic kidney disease or even nephrotic edema, but on the latter, there is only one RCT published so far [18,19,20,21]. Therefore, the aim of our study was to assess the non-inferiority and safety profile of the oral combination of furosemide/hydrochlorothiazide/amiloride compared to i.v. furosemide in patients with NS and resistant edema.

## 2. Materials and Methods

### 2.1. Study Design and Participants

We performed a prospective, randomized, single-center, non-inferiority study comparing the efficiency and safety of furosemide/hydrochlorothiazide/amiloride combination and i.v. furosemide in patients with RNE. The patients’ enrollment was initiated in January 2021 and was completed in December 2022. In the screening phase of the trial, we evaluated all patients over 18 years old with nephrotic syndrome and volume overload who were admitted to the Fundeni Clinical Institute, Nephrology Department. Medical history, physical examination and relevant laboratory data were gathered. 

A number of 22 patients with NS and resistant edema defined as hypervolemia with a sodium excretion fraction (FENa+) < 0.2% was included. All patients had an eGFR > 30 mL/min/1.73 m^2^.

The exclusion criteria were pediatric population, nephrotic syndrome in patients with type 1 or type 2 diabetes, eGFR < 30 mL/min/1.73 m^2^, hypokalemia and hyperkalemia (defined as a serum potassium < 3 mEq/L or > 6 mEq/L, respectively), severe hyponatremia (defined as a serum natrium < 125 mEq/L), uncompensated metabolic alkalosis (defined as a pH > 7.5, serum bicarbonate > 30 mEq/L without a resultant increase in the PaCO_2_ value), severe pulmonary congestion defined as dyspnea, orthopnea, rales and an abnormal oxygen saturation (SpO_2_) level of <90% with no oxygen, active infection, NSAIDs usage within one month prior to study enrollment, pregnancy, kidney transplant, known allergy to furosemide, hydrochlorothiazide or amiloride, and patients with a defibrillator/pacemaker/metal prosthesis.

The study was approved by the Ethics Committee of Fundeni Clinical Institute (No. 46275/8 September 2020) and was registered in the ISRCTN registry on December 2020 (https://www.isrctn.com accessed on 10 October 2023, ISRCTN number: 28785798). 

### 2.2. Study Protocol

#### 2.2.1. Randomization and Treatment

**Figure 1:**
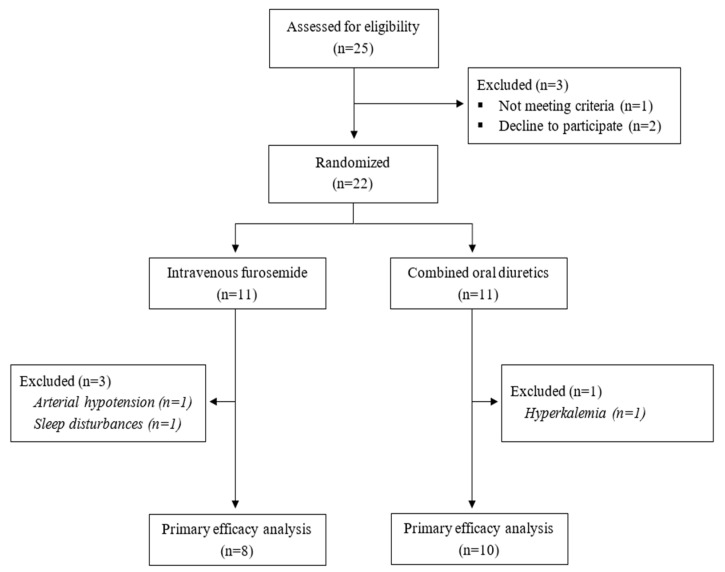
Study flow diagram.

Prior to randomization, all patients had a 24 h washout period during which no diuretics were administered. After that, patients were randomly assigned to one of the two treatment groups, as illustrated in Figure 1. Group 1 received intravenous furosemide, starting with a bolus of 40 mg, followed by continuous administration of 5 mg/h for 5 days, with dose adjustment according to urinary output (if urinary output >5 L/24 h—the dose was decreased to 2.5 mg/h; if urinary output <5 L/24 h—the same dose of 5 mg/h was maintained). Group 2 received oral furosemide at 40 mg/day and oral hydrochlorothiazide/amiloride at 50/5 mg/day for 5 days. We chose these doses based on other data, which showed treatment efficacy in volume overload from other causes (mostly heart failure). Oral medication was given separately, every day, in order to avoid overmedication. Both the patients and clinical investigators were aware of the type of medication. Immunosuppression was allowed during diuretic treatment because we considered it to be unethical to deprive patients with severe nephrotic syndrome of the correct treatment for a period of 5 days.

#### 2.2.2. Measurements

We evaluated patients at baseline and after randomization daily, for 5 days. Clinical and laboratory measurements were performed. We measured body weight with the same digital scale, urine output, systolic and diastolic blood pressure using a manual sphygmomanometer in a sitting position after 5 min of rest and hydration status by bioimpedance at baseline and at the end of the follow-up period. For bioimpedance, we used a body composition monitor (BCM). Laboratory measurements were serum markers (creatinine, urea, albumin, hematocrit, sodium, potassium, calcium, magnesium, bicarbonate and pH), urinary markers (creatinine and sodium for FENa at baseline) and 24-h sodium and potassium at baseline and daily after randomization during the follow-up period. Urine sodium and potassium were reported as the total amount of sodium and potassium excreted in urine in a 24-h period, calculated by multiplying urine sodium and potassium concentrations in an aliquot of a 24-h urine sample by 24-h urine volume.

### 2.3. Endpoints

The primary endpoint was the change in weight after 5 days of treatment. Secondary endpoints were hydration status change measured by bioimpedance, changes in urine output, systolic blood pressure, diastolic blood pressure, serum creatinine, serum urea, serum albumin, serum and urinary electrolytes, acid-base parameters and the safety profile (arterial hypotension defined as a systolic and diastolic blood pressure of <90/50 mmHg; severe hyponatremia defined as a serum sodium of <125 mEq/L; hypo- and hyperkalemia defined as a serum potassium of <3.0 and >6.0 mEq/L; severe hypomagnesemia defined as a serum magnesium of <1.0 mg/dL; uncompensated metabolic alkalosis defined as a pH > 7.5, serum bicarbonate >30 mEq/L without a resultant increase in the PaCO_2_ value; acute kidney injury defined according to KDIGO) [22].

### 2.4. Statistical Analysis

Continuous variables were expressed as mean ± SD and categorical variables as numbers and percentages. Differences for baseline characteristics were analyzed using Student’s *t*-test for continuous variables, and chi-square test and Fisher’s exact test were deemed appropriate for categorical variables. Mean change from baseline for continuous variables in each treatment group was obtained using a paired sample *t*-test. The mean change between the two groups for each continuous measured parameter was expressed as the adjusted mean difference with a 95% confidence interval (CI) and was performed using analysis of covariance (ANCOVA), considering baseline parameters as covariates. Statistical analysis and figures were performed with SPSS version 26 (SPSS Inc. Software, Chicago, IL, USA) and GraphPad Prism version 10.0.0 (1992–2023 GraphPad Software, LLC, San Diego, CA, USA). A *p* value < 0.05 was considered statistically significant.

## 3. Results

### 3.1. Baseline Characteristics

As noticed in Figure 1, 25 patients underwent screening and 22 were enrolled in the study. The patients were equally randomized between the two treatment groups. In the i.v. furosemide group, three patients presented adverse events (AE) and stopped the treatment, and in the oral diuretic combination group, one patient discontinued the treatment due to AE. Therefore, 18 patients, 8 in the i.v. furosemide group and 10 in the oral diuretic combination group, completed the trial and were included in the intention-to-treat analysis. 

Baseline characteristics are shown in Table 1. No statistically significant differences between the two groups were observed. However, patients from the i.v. furosemide group received less frequent immunosuppressive treatment at baseline (27.3 vs. 72.7%, *p* = 0.08) and had a lower mean hydration status measured by bioimpedance (5.46 ± 3.52 vs. 9.23 ± 4.71 L, *p* = 0.05), compared to patients from the oral furosemide/hydrochlorothiazide/amiloride group.

### 3.2. Primary and Secondary Endpoints Analysis

**Figure 2:**
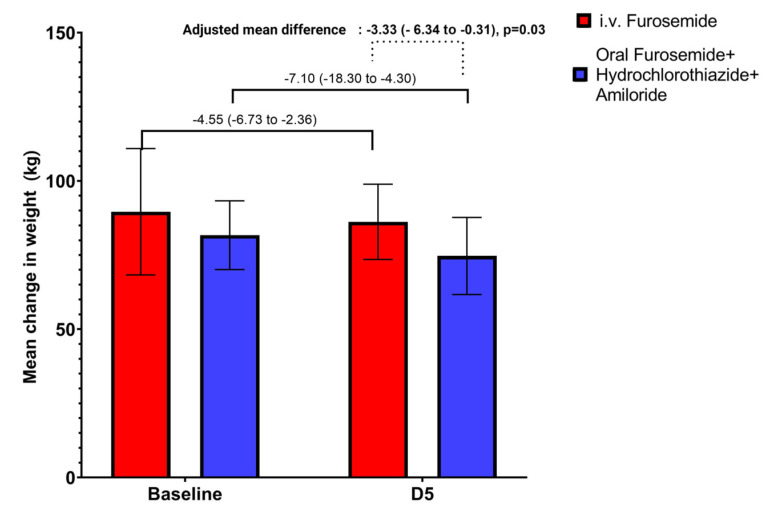
Mean change in weight from baseline to day 5 and the adjusted mean difference of weight between the two groups after 5 days of treatment.

Changes in analyzed parameters after 5 days of treatment are depicted in Table 2. The adjusted mean difference regarding change in weight from baseline between the two groups was favored by the treatment with combined diuretics [adjusted mean difference: −3.33 kg (95% CI: −6.34 to −0.31), *p* = 0.03] (Figure 2).

**Figure 3:**
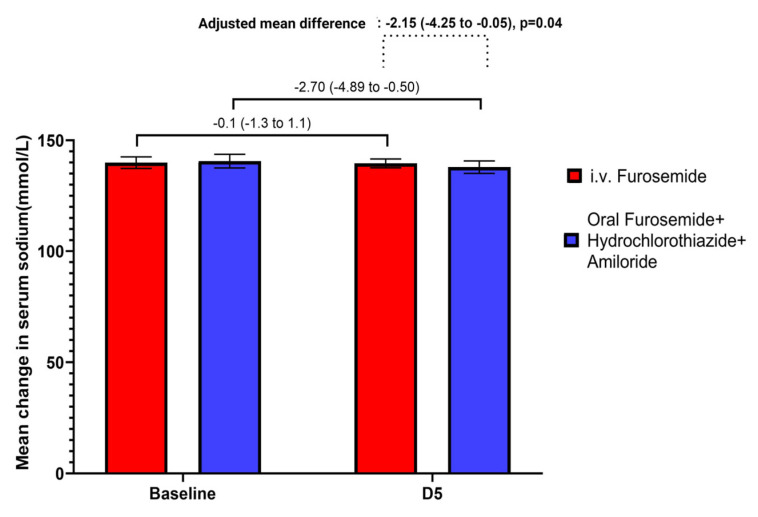
Mean change in serum sodium from baseline to day 5 and the adjusted mean difference of serum sodium between the two groups after 5 days of treatment.

There were no differences between groups regarding hypervolemia evaluated with bioimpedance, nor between systolic blood pressure, diastolic blood pressure, serum albumin, serum creatinine, BUN, serum calcium, serum magnesium, serum potassium, acid-base parameters, 24-h urine output and 24-h urinary sodium and potassium excretion. We observed a significant decrease in the adjusted mean difference of serum sodium of −2.15 mmol/L [(95% CI: −4.25 to −0.05), *p* = 0.04], favored by combined oral diuretic treatment [−2.70 mmol/L (95% CI: −4.89 to −0.50) vs. −0.10 mmol/L (95%CI: −1.30 to 1.10)]. No cases of hyponatremia <130 mEq/L were found (Figure 3).

### 3.3. Adverse Events Analysis

Regarding adverse events, no significant differences were observed between groups. Nine adverse events were identified in each group of treatment; 27.3% of patients stopped treatment due to AE in the i.v. furosemide group and 9.1% in the oral combination group (*p* = 0.58). The most common adverse reaction observed in the two groups was metabolic alkalosis, which was seen in four patients (36.4%) in the i.v. furosemide group and in five patients (45.5%) in the oral diuretic combination group (Table 3).

## 4. Discussion

As far as we know, this is the only randomized clinical trial evaluating amiloride in patients with nephrotic syndrome and edema and proving its efficacy and safety in association with oral furosemide and hydrochlorothiazide compared to intravenous furosemide alone. We showed that oral diuretic combination had a significant impact on weight change in the first 5 days after treatment initiation. This finding is in concordance with another study of eight patients with severe diuretic resistant edema, five of whom presented with nephrotic syndrome, which tested the effect of amiloride, chlorothiazide, ethacrynic acid and furosemide, independently or in combination. Amiloride was half to one-third as potent as the other diuretics. It was concluded that it was a mild diuretic and acted additively when used in combination with other diuretics [23]. We observed no significant difference regarding hydration status between the two groups after 5 days of treatment. There could be some possible explanations as to why patients from the oral diuretic combination group achieved a significant reduction in weight loss but not in the hydration status. Apart from a lower hypervolemia status in the i.v. furosemide group at baseline, an issue regarding bioimpedance measurement and timing could be involved. Patients on oral diuretic combination had a rapid and significant response to the diuretic, which was reflected in their weight. The fact that this was not reflected in the hydration status can be explained by the extra- and intracellular fluid distribution. Bioimpedance mainly measures extracellular fluid levels. Diuretics primarily act on extracellular fluid, so changes in total body water may not be as significant. Moreover, bioimpedance measurements might have not captured rapid changes in hydration status that occurred after effective diuretic treatment in nephrotic syndrome, such as oral diuretic combination with furosemide, hydrochlorothiazide and amiloride. Thus, it might take some time to reach equilibrium and the redistribution of fluids after diuretic treatment. A longer period of follow-up might have clarified this conundrum. 

There is only one clinical trial evaluating diuretic efficacy in nephrotic edema that has been conducted. It was a randomized, small-sized study of 20 patients with nephrotic resistant edema, which demonstrated the benefit of acetazolamide and hydrochlorothiazide followed by furosemide therapy compared to furosemide and hydrochlorothiazide followed by furosemide. The first diuretic combination showed a significantly greater mean weight decrease and 24-h urine volume increase. The authors underlined the role of pendrin, a Cl-/HCO_3_- exchanger in the intercalated cell of the collecting duct, in compensatory distal tubule salt reabsorption when NCC is inactivated by hydrochlorothiazide. Therefore, the downregulation of pendrin by acetazolamide and the inhibition of NCC at the same time can generate significant diuresis [24]. But nephrotic syndrome is a hypervolemic state where fractional proximal tubular sodium reabsorption is low, unlike heart failure or cirrhosis, with a lower response when using acetazolamide. Consequently, we believe that amiloride is a better choice of treatment. Even though relevant data do not exist, except animal studies and case reports, ENaC is considered the dominant pathway of diuretic resistance in nephrotic edema, and so, amiloride, an ENaC-mediated diuretic, appears to be an optimal management option, especially when used with other diuretics. To this day, there are no large clinical trials to support the use of amiloride in nephrotic edema, only few data and case reports that show a better or unclear treatment option using amiloride [25,26]. Even though we did not include patients with diabetes mellitus, it is worth mentioning a few studies on the diabetic population that convey contradictory information about amiloride. 

Studies have demonstrated that ENaC plays an important role in sodium and water retention in any disorder with proteinuria (chronic kidney disease, heart failure, preeclampsia or diabetic nephropathy). Many proteases have the ability to activate ENaC (prostasin, transmembrane protease serine 4—TMPRSS4, matriptase, cathepsin B, elastase and kallikrein), but plasmin remains the most important. It is the active form of plasminogen resulting mostly from the action of uPA [27,28,29,30,31]. So, it is understandable why amiloride would be effective. A pilot double-blind randomized cross-over study compared amiloride to hydrochlorothiazide in terms of safety and efficacy in patients with type 2 diabetes and macroscopic proteinuria. The trial was stopped early because of safety concerns. In the amiloride group, patients developed severe hyperkalemia and acute kidney injury, even though the study enrolled only patients with eGFR > 60 mL/min/1.73 m^2^. Based on the collected date until the study was ceased, there were no significant differences in systolic blood pressure or weight, but a better control in blood pressure in patients with high level of urinary plasminogen taking amiloride was noticed. Possible explanations for the adverse effects could have been the association of antihypertensive medications, such as an angiotensin-converting enzyme inhibitor or angiotensin receptor blocker, the fact that doses of 10 or 20 mg of amiloride could have been too high and maybe lowered doses of 5 mg/d could have had a better safety profile [32]. Anderson et al. demonstrated in a study on type 1 diabetic patients with or without (controls) diabetic nephropathy that amiloride increased significantly the total and fractional sodium excretion in both groups, but natriuresis and weight loss were larger in the control group. They concluded that the negative outcome should be interpreted with respect to study power and the possibility of false rejection of the hypothesis, due to not having a healthy control group or placebo treatment [33].

Referring to patients with nephrotic syndrome, there are two case reports which indicated the benefit of amiloride. The first case report described the case of a 38 y.o man with nephrotic edema related to type 1 diabetes mellitus who was started on intravenous furosemide at 80 mg/day, with increased doses up to 160 mg/day, and still with no response. The patient was already on thiazide and spironolactone, started on a previous clinical visit. Therefore, a low dose of 5 mg/day of amiloride was initiated with a decrease of 7 kg for the next 2 weeks. He also experienced hyperkalemia, which resolved after stopping both spironolactone and amiloride. Even more so, after a period of 5 weeks, he was started again on amiloride with no hyperkalemia this time [34]. The second case report presented a 55 y.o man with nephrotic syndrome secondary to hepatitis C virus-related cryoglobulinemic membranoproliferative glomerulonephritis who was admitted to hospital with severe weight gain. Intravenous loop diuretic was started, but the patient did not respond well, so a combination of loop diuretic and triamterene was tried, which effectively reduced edema [35].

As mentioned before, the issue of treatment with amiloride is the risk of hyperkalemia. In our study, there was no statistically significant difference in serum potassium between the two treatment groups, although there was a slight increase in serum potassium in the amiloride group (one patient had severe hyperkalemia leading to treatment withdrawal). Previous reports showed similar data. [23,36]. Another electrolyte disturbance found in the oral diuretic combination group in our study was a significant mean decrease in serum sodium, but none of the patients experienced severe hyponatremia. This could be explained by the sequential nephron blockade associating a greater risk of hyponatremia, as this group of patients also had an increased 24-h urine sodium, even though it was not an increment of statistical significance. [1,37,38]. Regarding other adverse events that lead to medication withdrawal, arterial hypotension (18.2%) was observed in the i.v. furosemide group only. This could be explained by a lower hypervolemic state at baseline compared to patients in the oral diuretic combination group and by the etiology of nephrotic syndrome in this group. One patient had AA amyloidosis, which is a rare disease that manifests with low blood pressure [39].

The limitations of this study are its small sample size, short follow-up, lack of blinding, the difference in hypervolemic state between the two groups at baseline and the fact that 72.8% of patients treated with oral diuretics benefited from immunosuppressive treatment, while only 28.6% of patients with intravenous furosemide had immunosuppression. It is not excluded that the two baseline differences might have contributed to the treatment response, and the results should be interpreted in this context. Future research may consider larger populations and longer follow-up periods for a more comprehensive assessment. 

## 5. Conclusions

In conclusion, we have demonstrated that oral diuretic combination of furosemide, hydrochlorothiazide and amiloride was non-inferior to i.v. furosemide in terms of weight control of patients with RNE and a similar safety profile.

## Figures and Tables

**Table 1:** Baseline characteristics between the two groups.

	i.v. Furosemide Group (*n* = 11)	Oral Furosemide + Hydrochlorothiazide + Amiloride Group (*n* = 11)	*p* Value
Age (mean, years)	49 ± 11.35	46.55 ± 20.09	0.72
Gender (%)			0.40
Male	4 (36.4%)	6 (54.5%)	
Female	7 (63.6%)	5 (45.5%)	
Nephrotic syndrome etiology (%)			0.10
MN	4 (36.4%)	6 (54.5%)	
FSGS	1 (9.1%)	0 (0%)	
MCD	2 (18.2%)	3 (27.3%)	
LN	0 (0%)	2 (18.2%)	
Other	4 (36.4%)	2 (18.2%)	
Nephrotic syndrome type (%)			1
New onset	10 (90.9%)	9 (81.9%)	
Relapse	1 (9.1%)	2 (18.2%)	
Previous immunosuppression (%)	1 (9.1%)	3 (27.3%)	0.58
Current immunosuppression (%)	3 (27.3%)	8 (72.7%)	0.08
Current immunosuppression type (%)			0.11
GC	1 (9.1%)	3 (27.3%)	
CYC	1 (9.1%)	0 (0%)	
CsA	0 (0%)	2 (18.2%)	
RTX	1 (9.1%)	1 (9.1%)	
CYC, GC	0 (0%)	1 (9.1%)	
CYC, RTX, GC	0 (0%)	1 (9.1%)	
Without	8 (72.7%)	3 (27.3%)	
Weight (mean, kg)	89.6 ± 21.27	81.79 ± 11.60	0.30
Urine output (mean, mL/24 h)	1263.64 ± 520.14	1145.45 ± 598.93	0.62
Hydration status (mean, L)	5.46 ± 3.52	9.12 ± 4.71	0.05
Systolic BP (mean, mmHg)	125.45 ± 31.10	128.63 ± 19.50	0.77
Diastolic BP (mean, mmHg)	79.09 ± 10.44	82.72 ± 12.72	0.47
Serum creatinine (mean, mg/dL)	1.09 ± 0.48	1.22 ± 0.59	0.59
Serum urea (mean, mg/dL)	38.70 ± 18.49	47.39 ± 14.47	0.23
Serum albumin (mean, g/dL)	2.18 ± 0.32	1.80 ± 0.75	0.13
pH	7.38 ± 0.03	7.38 ± 0.04	1
Serum sodium (mean, mmol/L)	141.02 ± 2.70	138.04 ± 3.34	0.50
Serum potassium (mean, mmol/L)	4.34 ± 0.35	4.16 ± 0.60	0.40
Serum calcium (mean, mg/dL)	7.99 ± 0.53	7.98 ± 0.64	0.97
Serum magnesium (mean, mg/dL)	1.73 ± 0.34	2.06 ± 0.32	0.17
Serum bicarbonate (mean, mmol/L)	30.42 ± 2.71	28.57 ± 3.77	0.44
HTC (mean, %)	34.03 ± 7.29	37.63 ± 3.36	0.15
24 h urine measurements			
Sodium (mean, mmol/L)	54.25 ± 29.79	58.59 ± 35.32	0.75
Potassium (mean, mmol/L)	25.98 ± 8.01	36.73 ± 20.34	0.11
Proteinuria (mean, g/24 h)	7.04 ± 3.50	6.91 ± 2.73	0.92

*n* = number; MN = membranous nephropathy; FSGS = focal segmental glomerulonephritis; MCD = minimal change disease; LN = lupus nephritis; GC = glucocorticoids; CYC = cyclophosphamide; CsA = ciclosporine; RTX = rituximab.

**Table 2:** Intention-to-treat analysis regarding change from baseline to day 5 between the two groups.

	Oral Furosemide + Hydrochlorothiazide + Amiloride Group	i.v. Furosemide Group		
	Mean Change from Baseline (95% CI)	Mean Change from Baseline (95% CI)	Adjusted Mean Difference (95% CI)	*p* Value
Δ Weight (kg)	−7.10 (−18.30 to −4.30)	−4.55 (−6.73 to −2.36)	−3.33 (−6.34 to −0.31)	0.03
Δ Hydration status (L)	−4.71 (−6.87 to −2.54)	−3.91 (−5.69 to −2.13)	−0.05 (−2.60 to 2.60)	0.96
Δ Systolic BP (mmHg)	−7.50 (−20.25 to 5.25)	−3.12 (−27.89 to 21.65)	−2.25 (−17.17 to 12.67)	0.73
Δ Diastolic BP (mmHg)	−6.50 (−15.10 to 2.10)	−8.75 (−19.17 to 1.67)	−5.64 (−13.14 to 1.86)	0.13
Δ Serum creatinine (mg/dL)	0.23 (0.11 to 0.36)	0.21 (−0.03 to 0.46)	0.03 (−0.18 to 0.26)	0.73
Δ Serum urea (mg/dL)	17.49 (6.5 to 28.47)	10.32 (−5.26 to 25.91)	7.87 (−9.50 to 25.25)	0.35
Δ Serum albumin (g/dL)	0.60 (0.11 to 1.08)	0.21 (−0.31 to 0.74)	0.14 (−0.47 to 0.76)	0.62
Δ pH	0.02 (−0.01 to 0.07)	0.05 (0.02 to 0.08)	−0.01 (−0.06 to 0.03)	0.42
Δ Serum sodium (mmol/L)	−2.70 (−4.89 to −0.50)	−0.10 (−1.30 to 1.10)	−2.15 (−4.25 to −0.05)	0.04
Δ Serum potassium (mmol/L)	0.11 (−0.27 to 0.51)	−0.35 (−0.66 to −0.05)	0.38 (−0.11 to 0.87)	0.11
Δ Serum calcium (mg/dL)	0.12 (−0.36 to 0.6)	−0.1 (−0.51 to 0.31)	0.27 (−0.33 to 0.87)	0.35
Δ Serum magnesium (mg/dL)	−0.03 (−0.28 to 0.22)	−0.02 (−0.38 to 0.34)	0.22 (−0.01 to 0.45)	0.06
Δ Serum bicarbonate (mmol/L)	2.50 (−0.30 to 5.30)	1.92 (−2.39 to 6.24)	0.21 (−4.41 to 4.83)	0.92
Δ HTC (%)	1.17 (−1.97 to 4.31)	0.25 (−2.08 to 2.58)	0.83 (−3.39 to 5.06)	0.68
Δ 24 h measurements				
Urine output (mL/24 h)	1090 (418.55 to 1761. 44)	1087.5 (−187.01 to 2362.01)	7.78 (−1277.41 to 1292.97)	0.99
Sodium (mmol/L)	26.79 (2.84 to 50.74)	16.04 (−19.75 to 51.83)	12.94 (−19.21 to 45.1)	0.40
Potassium (mmol/L)	−14.93 (−29.28 to −0.58)	−8.41 (−15.89 to −0.93)	−1.76 (−12.76 to 9.22)	0.73
Proteinuria (g/24 h)	0.06 (−4.17 to 4.30)	−0.17 (−6.49 to 6.14)	−0.49 (−6.35 to 5.36)	0.85

Δ = mean change; CI = confidence interval; HTC = hematocrit.

**Table 3:** Safety profile.

	i.v. Furosemide Group (*n* = 11)	Oral Furosemide + Hydrochlorothiazide + Amiloride Group (*n* = 11)	*p* Value
Total AE number	9	9	-
Worsening hypervolemia	1 (9.1%)	0 (0%)	1
Hypotension	2 (18.2%)	0 (0%)	0.47
Severe hyperkalemia	0 (0%)	1 (9.1%)	1
Metabolic alkalosis	4 (36.4%)	5 (45.5%)	1
AKI	2 (18.2%)	3 (27.3%)	1
Medication withdrawal due to AE	3 (27.3%)	1 (9.1%)	0.58
Severe hyperkalemia	0 (0%)	1 (9.1%)	
Arterial hypotension	2 (18.2%)	0 (0%)	
Sleep disturbances	1 (9.1%)	0 (0%)	

i.v. = intravenous; *n* = number; AE = adverse events; AKI = acute kidney injury.

## Data Availability

The datasets generated and/or analyzed during the current study are available from the corresponding author on reasonable request.
